# Association of 5-lipoxygenase activating protein gene polymorphism and stroke: A study from north east of Iran

**Published:** 2019-07-06

**Authors:** Marjan Erfani, Ariane Sadr-Nabavi, Reza Jafarzadeh-Esfehani, Mohammad Shariati, Leila Ghanbari-Garekani, Samaneh Vojdani-Chahchaheh, Payam Sasannejad

**Affiliations:** 1 Department of Neurology, School of Medicine, Mashhad University of Medical Sciences, Mashhad, Iran; 2 Department of Medical Genetics, School of Medicine, Mashhad University of Medical Sciences, Mashhad, Iran; 3 Department of Genetics, Shahid Beheshti University of Medical Sciences, Tehran, Iran

**Keywords:** Stroke, Polymorphism, Genetic, Polymerase Chain Reaction, Restriction Fragment Length

## Abstract

**Background:** Stroke is a multifactorial disorder and a major cause of morbidity and mortality around the world. There are growing numbers of candidate gene pathways which are thought to be associated with stroke. Genes involved in lipid metabolism are important issues in stroke studies. Studying different polymorphisms in these genes are becoming an interest for researchers. 5-lipoxygenase activating protein (ALOX5AP) is one of these genes. Different studies have provided different relations between ALOX5AP promoter polymorphism (rs17222919) and stroke. In the present study, we have evaluated this gene polymorphism in a population in north east of Iran.

**Methods:** This case-control study took place in Ghaem Hospital, Mashhad, Iran. Patients with computed tomography (CT) or magnetic resonance imaging (MRI) confirmation for ischemic stroke were enrolled in this study and considered as case group. Healthy persons without ischemic stroke were control group. During 1-year period of this study, ALOX5AP gene polymorphism in 200 healthy patients (control group) as well as 228 patients with stroke (case group) was evaluated by polymerase chain reaction-restriction fragment length polymorphism (PCR-RFLP).

**Results:** All of 428 persons (228 cases and 200 healthy controls) enrolled in this study. The genotype and allele frequency was significantly different between both groups (P = 0.001 and P = 0.003, respectively). A total number of 54 patients had G allele in case group in contrast to 27 ones in control group. Also, 174 patients in case group had T allele and 173 persons had this allele in control group. In compression of TT genotype, the risk of developing stroke in GG and TG genotypes increased by 3.998 and 1.643, respectively. Also the risk of ischemic stroke with G allele would increase by 2.128.

**Conclusion:** According to our results, ALOX5AP promoter polymorphism (rs17222919) is related to increased ischemic stroke in Iranian population.

## Introduction

Stroke is a well-known cause of death which has evoked global concern with different regional prevention programs. Ischemic stroke is more prevalent than hemorrhagic stroke, ranging from 62% to 87.0% in our country, Iran.^1^^-^^3^ The incidence is considerably greater than most western countries and also occurs in younger population. As same as global reports, among 2 major types of stroke, ischemic stroke has higher incidence than hemorrhagic stroke in our region.^4^^,^^5^ While there is not any remarkable difference between male and female population, hemorrhagic stroke is more likely to occur in female population in our region.^1^ Although stroke incidence, prevalence, and mortality have been dropped recently, but the overall burden has increased across the globe in both genders in every ages.^5^ Iran as a developing country faces many challenges for improvement of stroke care. In addition to insufficient equipment and stroke-ready canters, lack of public awareness about stroke management, symptoms, and prevention is a major concern in our country.^6^ Hypertension (HTN), hyperlipidemia, obesity, diabetes mellitus (DM), oral contraceptives, smoking, and heart disease are some of the most common risk factors for stroke. Among these risk factors, HTN and history of previous stroke are major risk factors of stroke in our population.^7^ According to Mashhad stroke and heart atherosclerotic disorder (MASHAD) study, 88.4% and 79.2% of female and male population had at least one lipid abnormality and other cardiovascular risk factors were also higher than western countries.^4^ Besides these traditional risk factors, recently genetic risk factors are also becoming more prominent. There are many polymorphisms reported as risk factors for different types of stroke. Genetic variations in matrix deposition, inflammation, and lipid metabolism are considered to be associated with increased risk of both ischemic and hemorrhagic stroke. Effective genes in lipid metabolism such as paraoxonase/arylesterase 1 (PON1), cholesteryl ester transfer protein (CETP), apolipoprotein E (APOE), and 5-lipoxygenase activating protein (ALOX5AP) are subjects of recent studies about stroke.^8^^,^^9^ Among these genes, ALOX5AP has received the most attention. Variations in this gene are becoming an interest for researchers as predictors of the risk of vascular diseases. ALOX5AP gene product is a mediator of leukotrienes and responsible for accumulation of these leukotrienes in fatty deposits on arterial walls. This accumulation will be a target for immune system and therefore, atherosclerosis may develop.^9^ However, the exact mechanism of developing cerebrovascular accident (CVA) and polymorphisms of ALOX5AP is not clear. Some studies have reported an association between ALOX5AP promoter polymorphism (rs17222919) and stroke.^9^^-^^11^ While stroke is a multifactorial and polygenic disease, studying such polymorphisms along with other traditional risk factors is useful for establishing life style modifications as well as predicting patient’s response to treatment and outcomes. The present study has been conducted in order to evaluate the relation between genetic polymorphism of ALOX5AP gene and developing ischemic stroke in north east of Iran.

## Materials and Methods

This one-year case-control study has been approved by Ethics Committee of Mashhad University of Medical Sciences, Mashhad, Iran, and took place in Ghaem Hospital, Mashhad City. Patients who were admitted in stroke intensive care unit (ICU) or neurology emergency ward and had first-time stroke were candidates to enroll in this study. After taking an informed consent, according to the two independent proportions formula and as same as our previous study,^12^ with the power of 0.8, P1: 0.18 and P2: 0.3; the sample size was calculated as 200 patients in case and control groups.


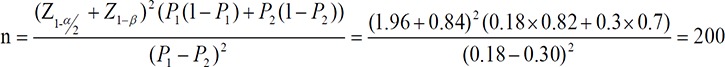


***Formula:*** The diagnosis of stroke in case group was made by a neurologist and confirmed by imaging studies such as brain computed tomography (CT) or brain magnetic resonance imaging (MRI). Pregnant women and patients with prior history of head trauma or surgery were excluded. After filling an informed consent form, 5 ml of participants’ peripheral blood was taken in ethylenediaminetetraacetic acid (EDTA) tubes and stored in -20 °C. The samples were transferred to genetic department of Mashhad University of Medical Sciences for deoxyribonucleic acid (DNA) extraction. After extracting DNA by salting out method, amplification of coding exon was done by polymerase chain reaction (PCR). The primer pairs were designed using Primer3 Online. PCR was performed by the Labcycler (SensoQuest, Germany) thermocycler and amplification conditions were as follows: 94 °C for 5 minutes, followed by 35 cycles of 94 °C for 30 seconds, 60-68 °C for 30 seconds, and 72 °C for 30 seconds. Then, 72 °C was applied for 5 minutes as final extension. Reaction products were digested with Ddel restriction endonuclease [New England Biolabs (NEB)] during 1 hour at 37 °C. Results were observed in 3% agarose gel stained with green viewer. Fragment sizes of 177 bp and 79 bp indicated a wild-type homozygous TT genotype, and an uncut fragment of 223 bp indicated the homozygous GG genotype. The presence of all the three bands (177 bp, 79 bp, and 223 bp) indicated a heterozygous GT genotype. 

The study data were analyzed by SPSS software (version 21, IBM Corporation, Armonk, NY, USA). Distribution of quantitative data was evaluated by Kolmogorov-Smirnov test (K-S test). Distribution of qualitative data between groups was evaluated by chi-square test. T-test and Mann-Whitney U test were used for average comparison of quantitative data for normally and non-normally distribution, respectively. The relation between genotypes and allele in the study groups was evaluated by chi-square test. For the regression analysis, the P-value, odds ratio (OR), and 95% confidence interval (CI) for OR were stated.

## Results

**Figure 1:**
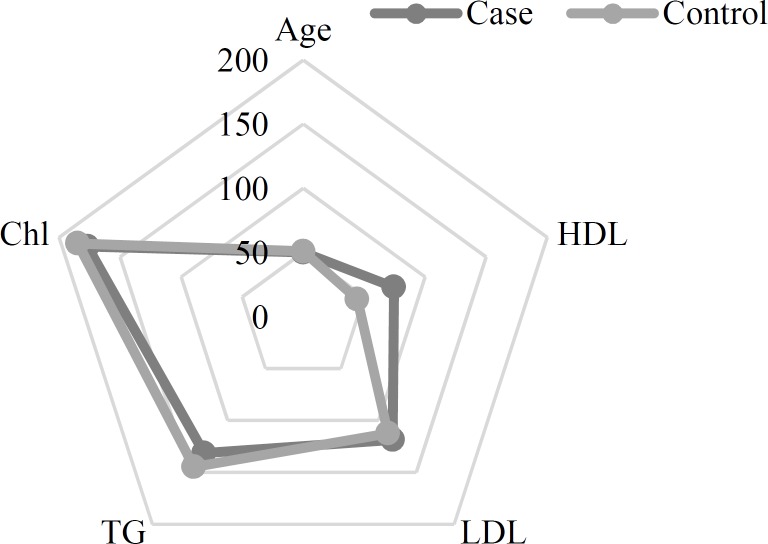
Distribution of risk factors between cases and controls

HDL: High-density lipoprotein; LDL: Low-density lipoprotein; TG; Triglyceride; Chl: Cholesterol

Among 428 participants, 218 were men and 210 were women. The case and control groups consisted of 228 and 200 participants, respectively; demographic data of these patients is summarized in table 1 and figure 1. 

HTN and cigarette smoking were significantly higher in case group (P < 0.001 and P = 0.005, respectively), while the gender and DM were not significantly different between both groups (P = 0.250 for both). Also, high-density lipoprotein (HDL), cholesterol, and triglyceride (TG) were significantly higher in case group (P < 0.001, P = 0.010, and P = 0.030, respectively), while age and low-density lipoprotein (LDL) were not significantly different between study groups (P = 0.530 and P = 0.510, respectively). The genotype and allele frequency of G and T alleles is shown in table 2.

**Table 1:** Demographic features of study participants

**Variable**		**n (%)**
Gender	Men	218 (50.1)
	Women	210 (49.1)
DM		105 (24.5)
HTN		195 (45.6)
Cigarette smoking		106 (24.8)
Type	Case	228 (53.3)
	Control	200 (46.7)
ALOX5AP	GG	33 (7.7)
	GT	96 (22.4)
	TT	299 (69.9)

DM: Diabetes mellitus; HTN: Hypertension; ALOX5AP: 5-lipoxygenase activating protein

The genotype and allele frequency was significantly different between both groups (P = 0.001 and P = 0.003, respectively). In compression of TT genotype, the risk of developing stroke in GG and TG genotypes increased by 3.998 fold and 1.643 fold, respectively.

The risk of developing stroke was also decreased by 0.470 in presence of T allele, while the G allele would increase the stroke risk by 2.128.

**Table 2:** Summary of genotype and allele frequency of G and T alleles in study groups

	**Case**		**χ²**	**P**	**Control**		**χ²**	**P**
	**Men [n (%)]**	**Women [n (%)]**			**Men [n (%)]**	**Women [n (%)]**		
G	29 (23.8)	25 (23.6)	0.002	0.960	12 (12.5)	15 (14.4)	0.320	0.540
T	93 (76.2)	81 (76.4)			84 (87.5)	89 (85.6)		
GG	12 (5.3)	13 (5.7)	0.980	0.610	3 (1.5)	5 (2.5)	0.390	0.820
TG	34 (14.9)	24 (10.5)			18 (9.0)	20 (10.0)		
TT	76 (33.3)	69 (30.3)			75 (37.5)	79 (39.5)		

The study risk factors (HTN, cigarette smoking, and DM) were not significantly related to allelic distribution (P = 0.300, P = 0.110, and P = 0.140, respectively), while higher LDL and HDL were significantly seen with T allele (P = 0.010 and P = 0.040, respectively).

## Discussion

Single nucleotide polymorphisms (SNPs) are known reason of variability between individuals which may cause susceptibility of different populations to various diseases. According to our results, ALOX5AP promoter polymorphism (rs17222919) is significantly related to increased risk of ischemic stroke. To the best of our knowledge, this is the first genetic study about the susceptibility of ALOX5AP gene polymorphism to ischemic stroke in Iranian population. Fan et al. have conducted a similar study on two independent cohorts. They have demonstrated that functional variation in ALOX5AP promoter (rs17222919) could significantly reduce the risk of ischemic stroke in Chinese population.^9^ Wang et al. study which was conducted on eastern Chinese populations showed that this SNP in ALOPX5AP was related to stroke in an opposite way in contrast to our study. They found that the TG genotype was significantly associated with increased risk of ischemic stroke in comparison with TT genotype. These patients reported to have 1.40 fold higher chance of developing small vessel stroke.^10^ Yang et al. study showed that G allele (rs17222919) frequency was significantly lower in Han Chinese ischemic stroke patients than control group. This study has selected 18 SNPs in ALOX5AP promoter region and showed that 6 loci were not polymorphic in their region. Also, other 11 SNPs which were in Hardy-Weinberg equilibrium (HWE) were not significantly related to ischemic stroke. Only the G allele frequency of rs17222919 was related to stroke.^11^

Another study which has evaluated the effect of rs17222919 polymorphism was conducted on Korean population. This study has successfully found a relationship between this SNP and intracranial hemorrhage (ICH), but not with ischemic stroke.^13^ These different results from different regions and even different time periods resemble in some key points. First of all, different methodology in each study seems to play the major role in achieving different results. Different sample sizes, SNP detection methods, statistical tests, and inclusion criteria are 4 major variations in similar studies about the relation of ALOX5AP and stroke. Second reason could be the differences between populations and sampling issues. For some populations, as same as ours, picking a group of patients who are perfectly matched according to their risk factors is not a simple issue. Also, designing two study groups which are in HWE is not simply possible in our population while migration and population size are two main issues for avoiding disequilibrium. The third issue could be other SNPs in the promoter region of ALOX5AP gene. Our study has only evaluated one of the main SNPs in this region, while other effective SNPs are also present. Although not all of these SNPs were related to ischemic stroke in other populations,^11^ however, they could be an effective predictor of stroke in our population and could be the subject of future studies. As a matter of fact, evaluating ALOX5AP polymorphism in different populations seems necessary to establish regional guidelines about genetic risk factors for developing stroke.

## Conclusion

According to our results, ALOX5AP polymorphism is associated with increased risk of ischemic stroke in our population. By knowing this fact, health care providers can focus their prevention programs on the patients with such polymorphisms in order to reduce other stroke risk factors and plan appropriate follow-up plans.

## Notes:

**How to cite this article:** Erfani M, Sadr-Nabavi A, Jafarzadeh-Esfehani R, Shariati M, Ghanbari-Garekani L, Vojdani-Chahchaheh S, et al. Association of 5-lipoxygenase activating protein gene polymorphism and stroke: A study from north east of Iran. Iran J Neurol 2019; 18(3): 114-8.
